# Impact of Intrinsic and Extrinsic Factors on the Pharmacokinetics of Peptides: When Is the Assessment of Certain Factors Warranted?

**DOI:** 10.3390/antib11010001

**Published:** 2021-12-21

**Authors:** Iftekhar Mahmood, Mark Pettinato

**Affiliations:** Mahmood Clinical Pharmacology Consultancy, LLC, Rockville, MD 20850, USA; pettinato@cua.edu

**Keywords:** peptides, pharmacokinetics, intrinsic and extrinsic factors, small molecule

## Abstract

Peptides are short chains of 2 to 50 amino acids (molecular weight of less than 10 kDa) linked together by peptide bonds. As therapeutic agents, peptides are of interest because the body naturally produces many different peptides. Short-chain peptides have many advantages as compared with long-chain peptides (e.g., low toxicity). The first peptide corticotropin was approved in 1952 for multiple inflammatory diseases and West syndrome. Since then, more than 60 peptides have been approved by the FDA. Pharmacokinetics (PK) is widely used in modern-day drug development for designing a safe and efficacious dose to treat a wide variety of diseases. There are, however, several factors termed as “intrinsic” or “extrinsic” which can influence the PK of a drug, and as a result, one has to adjust the dose in a patient population. These intrinsic and extrinsic factors can be described as age, gender, disease states such as renal and hepatic impairment, drug–drug interaction, food, smoking, and alcohol consumption. It is well known that these intrinsic and extrinsic factors can have a substantial impact on the PK of small molecules, but for macromolecules, the impact of these factors is not well established. This review summarizes the impact of intrinsic and extrinsic factors on the PK of peptides.

## 1. Introduction

Peptides are short chains of 2 to 50 amino acids (molecular weight of less than 10 kDa) linked together by peptide bonds [1]. A general structure of a peptide is R-CH(NH_2_)COOH, where NH2 is the amino group and COOH is a carboxyl group. When the carboxyl group of one amino acid reacts with another amino acid, a covalent bond is formed between the amino acid residues [1].

Peptides, polypeptides, and proteins are all chains of amino acids linked via peptide bonds. The terms “peptides” and “proteins” should not be confused. Not all peptides form proteins, but all proteins consist of peptides. Proteins are large peptides (polypeptides) containing 50 or more amino acids that consist of multiple peptide subunits. Structurally proteins are more complex than simpler peptides [1].

Peptides are short chains of amino acids (50 or fewer amino acids) whereas polypeptides and proteins are long chains and can be over 100 amino acids. The therapeutic peptides are generally under 40 amino acids in length [1].

As therapeutic agents, peptides are of interest because the body naturally produces many different peptides. Peptides are building blocks of hormones, toxins, proteins, enzymes, cells, and body tissues. Short-chain peptides have many advantages as compared with long-chain peptides. For example, structural simplicity, cost-effective synthesis on both small and large scales, high safety, low toxicity, low immunogenicity, and high binding affinity for a wide range of specific targets are the typical characteristics of peptides. The disadvantages are instability in blood or plasma, short half-life, and low or negligible oral bioavailability [1,2,3].

Peptides have several functions in the body and are also the basis of various medications. Peptides are mainly categorized as follows [2,3]:

- Number of amino acids making up the chain;
- Their source, animal or plant;
- Functions in the human body.

The first peptide, corticotropin, was approved in 1952. The route of administration was subcutaneous and the primary therapeutic area was central nervous system with approved indication for multiple inflammatory diseases and West syndrome. Since then, more than 60 peptides have been approved by the FDA [2].

Many peptides are currently being studied for use in treating different kinds of cancers. Research shows that atrial natriuretic peptide (ANP), for instance, can be potentially effective in treating colorectal cancer. Some peptide-based cancer treatments have already been approved and are being used to treat patients. Luteinizing hormone-releasing hormone (LH-RH) agonist drugs (also called GnRH agonist drugs), for example, are used to treat ovarian and prostate cancer [4].

Peptides play a special role in vaccines. Peptide-based vaccines mimic proteins that are naturally present in pathogens (germs that cause disease), allowing for certain responses to be replicated with what is usually a synthetic vaccine. In addition to providing immunity against specific pathogens, peptide-based vaccines are also used in cancer treatment; an anti-tumor T cell response is produced by vaccinating a patient with peptides from their tumor antigens [4,5].

Peptide-based vaccines hold much potential; however, they are not without their limitations. While the medical and scientific communities hope to develop an effective Alzheimer’s vaccine in the future, the difference between vaccines based on inactive or weakened pathogens and peptide-based vaccines is significant. Pathogen-based vaccines tend to cause a greater immune response which often leads to better protection [5].

Inflammation leads to several chronic diseases such as asthma, skin disorders, cancer, cardiovascular, arthritis, and neurological diseases. Many endogenous peptides showed anti-inflammatory characteristics by inhibiting or reducing the expression and activity of oxidants, cytokines, chemokines, and matrix metalloproteinases. Hence, peptides can be beneficial to provide relief or cure for inflammatory disease [6].

On 13 May 2021, the US Food and Drug Administration (FDA) sought comments on strategies for the pharmacological assessment of peptides, small polymers made up of 40 or fewer amino acids [7]. FDA was specifically interested in comments regarding the characterization of the effects of hepatic impairment, drug–drug interactions, and immunogenicity on the pharmacokinetics of peptides, as well as the effects of peptides on cardiac electrophysiology. The FDA pointed out that the development of peptides might involve other considerations; therefore, the FDA sought commentary in all relevant areas. The FDA request applies to peptides isolated from animal tissue as well as those produced through synthesis or a recombinant genetic process. The FDA pointed that although the Agency had been regulating peptides for decades, they did not have any regulatory and scientific guidance that specifically addresses considerations in clinical pharmacology recommendations for peptide development. Through their announcement on 13 May 2021, FDA sought input on when certain assessments are or are not warranted. These include looking at pharmacokinetics-based drug–drug interactions, pharmacokinetics in hepatic and renal impairment, immunogenicity and its downstream effects, and the potential for prolongation of the QT segment. Therefore, the objective of this review is to briefly describe the pharmacokinetics (PK) of peptides and discuss the impact of the aforementioned factors on the PK of peptides. The summary is based on the package inserts of approved peptides by the FDA and the European Medicines Agency (EMA). The last six years of package inserts were chosen because most of the current information about peptides is embedded in these package inserts.

## 2. Pharmacokinetics of Peptides

PK of peptides have been extensively reviewed by many authors [8,9,10,11,12,13]; therefore, in this review PK characteristics of peptides will be briefly described so that the readers can relate the PK of peptides with the aforementioned factors. The main focus of this review is on the evaluation of intrinsic and extrinsic factors affecting the PK of peptides.

### 2.1. Absorption

The peptides are generally given intravenously mainly because the peptides are unstable and extravascular routes of administration lead to low bioavailability. With a few exceptions such as cyclosporine A, most peptides have less than 1% oral bioavailability and have high intersubject variability in plasma [9]. Besides intravenous administration, peptides are also given subcutaneously (SC) or intramuscularly (IM). Following SC or IM administration, peptides enter into systemic circulation either through blood capillaries or the lymphatic system. Peptides with a molecular weight of 1–10 kDa are absorbed by both blood and lymphatic system [9]. Although subcutaneous or intramuscular administration avoids the degradation of peptides by hepatic or gastrointestinal enzymes, the degradation at the injection site due to protease or peptidase activity in the interstitial space and degradation in the lymphatic system for subcutaneous or intramuscular administration lead to lower bioavailability compared to intravenous administration [13,14,15].

The most convenient route of administration of a drug is oral. However, certain drugs cannot be administered by oral route mainly due to the instability of a drug in the gastrointestinal tract. Peptides face physiological and chemical/biochemical barriers in the gastrointestinal tract before being absorbed in the systemic circulation. These barriers include poor solubility in the gastrointestinal fluid, low permeability across the gastrointestinal mucosa due to their large size and hydrophilicity, the acidic nature of the gastric fluid, and secreted enzymes in the gastrointestinal fluids and nonsecreted enzymes in endothelial cells. All these physiological and chemical properties of the gastrointestinal tract result in the acidic hydrolysis and enzymatic degradation of many peptides [16,17,18,19]. All the aforementioned factors lead to poor bioavailability of oral peptides. Several peptides such as desmopressin, cyclosporin, semaglutide, taltirelin, linaclotide, and plecanatide are FDA-approved oral peptides [2]. Despite the poor bioavailability of oral peptides, efforts and new technology are being put forward to develop oral peptides. Besides oral and intravenous routes, peptides can also be administered by buccal, inhalational, intranasal, and transdermal routes [9,19,20]. Some approved drugs administered by intranasal and inhalational routes are lypressin (intranasal), calcitonin (intranasal), nafarelin (intranasal), and lucinactant (inhaled) [2].

### 2.2. Distribution

Since the size of peptides is between small molecules and macromolecules, diffusion and convection are both involved in the distribution of peptides [13]. The volume of distribution of peptides is small and limited to the extracellular space. The volume of distribution of the central compartment of peptides generally ranges from 3 to 8 L, slightly larger than blood volume [9]. The volume of distribution of peptides can be influenced by endogenous protein binding [9].

### 2.3. Metabolism or Degradation and Elimination of Peptides

In vivo, peptides are first metabolized to oligopeptides by endopeptidases, which cleave peptide bonds within the polypeptide chain. The oligopeptides are then further degraded to amino acids by exopeptidase (aminopeptidase or carboxypeptidase) which hydrolyzes peptides at the N- or C-terminus.

Peptides are broken down by a process called “proteolysis”. Proteolysis is typically catalyzed by cellular enzymes called proteases (also known as peptidases), but may also occur by intramolecular digestion. Luminally secreted enzymes such as pepsins, elastase, trypsin, and chymotrypsin play important roles in peptide proteolysis [21]. Proteolysis is a major elimination pathway for most peptides, and clearance of peptides can exceed cardiac output due to the degradation of peptides in blood [9].

Peptidases are widely distributed in the body, and blood, liver, kidneys, and small intestine are important sites for proteolytic degradation because these tissues contain a large number of various proteases and peptidases [9]. Other tissues such as lung, nasal epithelial cells, placenta, and skin also contain peptidases that can degrade peptides [22]. Degradation of peptides extensively takes place in blood, liver, kidneys, and gastrointestinal tract (GI) and is summarized below.

#### 2.3.1. Blood

The proteases are mainly distributed in the cytoplasm. Hydrophilic peptide drugs are generally hydrolyzed by soluble enzymes in the membrane and blood rather than by proteases. However, degradation of peptides by proteases in blood can also occur. For example, bradykinin, incretin GLP-1, and neuropeptide Y (NPY) are degraded by proteases in human plasma [23,24,25].

#### 2.3.2. Liver

The liver is an important site for peptide metabolism. In the liver, peptides are metabolized by transmembrane transport mediated by carriers and by endocytosis and pinocytosis [26]. Lipoprotein receptors found on the liver cell membrane may be responsible for the metabolism of plasminogen activators [27].

#### 2.3.3. Kidney

Peptides filtered by the glomerulus are hydrolyzed to amino acids by exopeptidases, endopeptidases, aminopeptidases, and carboxypeptidase found on the brush border membrane of the proximal tubule, and are then reabsorbed into systemic circulation by a specific amino acid transport system or degraded into small peptides and transported to proximal tubule epithelial cells. It was noted by Serada et al. [28] that teriparatide acetate was distributed and degraded in rat kidneys.

Since peptides are generally less than 10 kDa, their renal clearance may reach the glomerular filtration rate. Peptides can be filtered by the kidneys and then hydrolytic degradation in the brush border membrane of proximal tubule cells can take place [9].

#### 2.3.4. Gastrointestinal Tract

The degradation and metabolism of peptides by the GI tract is extensive, and as a result, oral administration of peptides is almost impossible. The GI tract contains a large number of proteases and peptidases. In the GI tract, peptides are degraded by three types of proteases [29]:

- Luminally secreted enzymes as well as pepsins, trypsin, chymotrypsin, elastase, and carboxypeptidase A/B;
- Brush border membrane-bound enzymes;
- Cytoplasmic enzymes.

Prodrug peptides can be metabolized to an active form. Linaclotide, a prodrug (14 amino acid peptide of the guanylin peptide family) is used for irritable bowel syndrome and is stable in the stomach. Linaclotide, however, can also be degraded to its metabolite MM-419447 following proteolysis and degradation [30].

Cytochrome P450 3A4 (CYP3A4) and P-glycoprotein (P-gp) are found at higher concentrations in the small intestine [31]. It has been well established that the metabolism of small molecules by intestinal CYP3A4 and P-gp can have an impact on the systemic bioavailability of orally administered drugs [31]. Not much is known regarding the effects of CYP3A and P-gp on peptides. Studies with the cyclic peptide immunosuppressant cyclosporine as well as peptidomimetics (a small protein-like chain designed to mimic a peptide, such as peptoids and β-peptides), the HIV-protease inhibitor saquinavir (Invirase), and a cysteine protease inhibitor K02 (Morpholine-Urea-Phe-Hphe-Vinyl sulfone) provide information on the impact of CYP3A4 and P-gp on the oral absorption of peptides [31].

Hepatic, renal, and biliary excretion of peptides may not be a major path of elimination of peptides [9]. However, there may be some exceptions (discussed later).

## 3. Strategies to Protect Peptides from Degradation (Proteolysis)

Over the years, several approaches have been taken to improve the PK of peptides by reducing their degradation process in the body. Some of these methods are summarized below [19,22].

Amino acid substitution or replacement of metabolically labile amino acids with unnatural or D-amino acids. Octreotide, GnRH antagonists and agonists, and ipamorelin are examples of amino acid substitution.

- Chemical modification of peptides at N- and/or C-terminus of a peptide. The chemical modification of a peptide can be achieved by N-acetylation and C-amidation. Tesamorelin is a hexenoyl moiety attached to the tyrosine residue at the N-terminus.
- Cyclization of linear peptides enhances the stability of these peptides in human serum. Furthermore, bicyclic peptides have higher proteolytic stability than the linear peptides and the cyclization of the peptide backbone improves stability.
- Nanoparticle formulations. Peptide-based nanomaterials consist of small peptide sequences that have a variety of properties. These nanomaterials have major advantages such as biocompatibility, high biological activity, bio-functionality, and easy modifiability. Peptide self-assembly is considered an effective method to improve the proteolytic stability of peptide drugs. Self-assembled peptide-based nanostructures such as tubes, filaments, fibrils, hydrogels, vesicles, and monolayers have been studied. Self-assembled lytic peptides have defined nanostructures and are protease-resistant. Nanostructures are developed from modified amino acids (N- or C-terminal modifications) to enhance cellular and in vivo stability. These modified nanostructures have shown enhanced drug delivery properties both under in vivo and in vitro conditions. Self-assembling peptides may be suitable for controlled release or targeting of anticancer drugs to tumor sites [22].
- Disulfide (DS) bridges help in the structural stabilization of peptides. Many peptides have more than one DS. Examples include lepirudin (65 amino acids, 3 DS), ziconotide (25 amino acids, 3 DS), calcitonin (32 amino acids, 1 DS), and linaclotide (14 amino acids, 3 DS). Three disulfide bridges make linaclotide stable enough for oral administration.
- Conjugation with polymers such as polyethylene glycol (PEG), conjugation to Fc antibody portion, and albumin fusion help in increasing the half-lives of peptides.

## 4. Impact of Intrinsic and Extrinsic Factors on the Pharmacokinetics of Peptides

The available pharmacokinetic data were used to determine if certain intrinsic and extrinsic factors are needed for the development of peptides as well as regulatory requirements for peptides. Intrinsic factors are those which are related to an individual. Age, gender, genetics, and disease states are examples of intrinsic factors. Extrinsic factors are the influence from outside. Examples include concomitant medicine (drug–drug interaction), food or beverages (alcohol), smoking, malnutrition, water deprivation, and environment. The intrinsic and extrinsic factors evaluated in this review are age (pediatrics, young, and elderly), sex and race, pregnancy, lactation, hepatic and renal impairment, drug interaction studies, and immunogenicity.

This review summarizes the impact of intrinsic and extrinsic factors on the PK of peptides. This review mainly focuses on the peptides approved by the United States Food and Drug Administration (US FDA), and most of the information was obtained from the FDA package inserts of the peptides. Due to the paucity of published literature on the clinical pharmacology aspects of peptides, the package inserts of the FDA were a good source of clinical pharmacology information. The approved package inserts of peptides by the FDA from 2015 to 2020 were assessed mainly because these have the latest information on the intrinsic and extrinsic factors [32,33,34,35,36,37,38,39,40,41,42,43,44,45,46,47]. There were 16 peptides (3 peptides as payloads in antibody–drug conjugates) for which evaluation was conducted. The following is a review of the impact of intrinsic and extrinsic factors on the PK of 16 peptides approved by the FDA. Peptides for diagnostic use were not included in this review. In Table 1, the indication and year of the approval of peptides are summarized. Table 2, Table 3 and Table 4 provide the summary of the impact of intrinsic and extrinsic factors on the PK of peptides.

## 5. Age (Elderly (65 Years and Older) versus Young) (Table 2)

All 16 peptides have descriptions of age. The impact of PK between young and elderly (>65 and <65 years of age) was reported. However, there were inconsistencies. In the majority of cases, no information was available on the impact of age (elderly 65 years or older). A general statement across the package inserts was “The effect of age 65 years or older on the pharmacokinetics is unknown”. Some package inserts indicated that the response rate and number of patients with a serious adverse event or safety and efficacy were similar between subjects >65 and <65 years of age. In some cases, it was reported that clinical studies did not include a sufficient number of subjects aged 65 and over to determine whether elderly patients respond differently from younger subjects.

## 6. Sex and Race (Table 2)

The overall conclusion was that sex and race had no clinically meaningful impact on the PK of peptides. For many peptides, no information was available in the FDA package insert.

## 7. Pediatrics (Table 2)

For 13 out of 16 peptides, no information on the PK of pediatrics was available in the FDA package insert. Based on the simulation from population pharmacokinetics, area under the curve (AUC) and maximum plasma concentration (C_max_) of Imcivree (setmelanotide) were 100% and 92% higher, respectively, in children 6 to <12 years of age as compared to patients greater than or equal to 17 years. For patients aged 12 to 17 years, the setmelanotide (AUC) and C_max_ were 44% and 37% higher, respectively, as compared to patients greater than or equal to 17 years. Based on the simulation results, FDA for pediatric patients aged 6 to <12 years suggested a starting dose of Imcivree of 1 mg injected subcutaneously once daily for 2 weeks (this is half of the dose given to 12 years of age and older).

Trulance (plecanatide) was contraindicated in pediatric patients <6 years of age. This was suggested based on the observation in young juvenile mice where plecanatide caused death due to dehydration. The PI suggested avoiding the use of Trulance in patients 6 years to <18 years of age because safety and efficacy of Trulance were not established in this age group.

According to FDA PI [43], after adjusting for body weight, the total exposure of Tresiba (insulin degludec) at steady state was found to be independent of age (1 to <18 years of age).

Biester et al. [48] conducted a randomized, single-dose PK study of insulin degludec (IDeg) in children (6–11 years, *n* = 12), adolescents (12–17 years, *n* = 13), and adults (18–65 years, *n* = 12) with type 1 diabetes. Subjects received a single subcutaneous dose of 0.4 U/kg IDeg. The results of the study indicated that AUC_0-__∞_ was higher in children compared to adults (children/adults ratio = 1.48) and in adolescents compared to adults (adolescents/adults ratio = 1.33). Ratios for C_max_ were 1.20 for children/adults and 1.23 for adolescents/adults. Population PK-based simulated steady-state AUC ratios were 1.52 for children/adults and 1.29 for adolescents/adults. Ratios for steady-state C_max_ were 1.50 for children/adults and 1.28 for adolescents/adults. This study indicated that the AUC of IDeg following a single dose and simulated AUC at steady state were approximately 50% higher in children than adults. However, neither the number of treatment-emergent hypoglycemic episodes nor severe hypoglycemic episodes were observed across age groups.

## 8. Renal Impairment (Table 3)

Like small molecules, for therapeutic peptides, the impact of renal impairment and end-stage renal disease (ESRD) on the PK and subsequently on the efficacy and safety of peptides requires evaluation.

For therapeutic proteins, in order to be eliminated by glomerular filtration, a threshold value of below 60 kDa is required [49]. Therefore, theoretically, one can assume that renal impairment may not have any impact on the PK of large proteins such as monoclonal antibodies, but one may observe the impact of renal impairment on the PK of smaller proteins and peptides below the cutoff point of 60 kDa such as interleukin-10, growth hormone, erythropoietin, and anakinra [49].

Considering the size of small peptides (40 kDa), it is anticipated that renal impairment will have an impact on the PK of peptides due to its elimination by glomerular filtration. The information gathered from the FDA package inserts (PIs) indicate that renal impairment studies were conducted for many peptides and the impact of the renal impairment was noted on the PK of many peptides. There were some peptides (*n* = 4) for which no information regarding renal impairment was available. The impact of renal impairment on the PK of peptides followed the same trend as of small molecules (the exposure is increased as a function of mild, moderate, or severe renal impairment as compared to subjects with normal renal function). In severe renal impairment, for some peptides, the AUC was generally 2-fold higher than normal renal function.

Generally, based on the magnitude of exposure in a disease state, the dose of a drug is adjusted. In the case of some peptides, no dose adjustment was recommended by the FDA in subjects with renal impairment despite the fact that the exposure of the particular peptide was much higher in subjects with renal impairment than in subjects with normal renal function. For example, a PK study indicated that C_max_ and AUC of Tymlos (abaloparatide) increased by 1.4- and 2.1-fold, respectively, in subjects with severe RI as compared to subjects with normal renal function. The FDA package insert indicated that no dosage adjustment is required for patients with mild, moderate, or severe renal impairment. Similarly, compared to healthy subjects, the C_max_ and AUC of Adlyxin (lixisenatide) increased by 60%, 42%, and 83% and 34%, 69%, and 124% in subjects with mild, moderate, and severe renal impairment, respectively. No dose adjustment in patients with RI was recommended. The reason for not adjusting the dose of abaloparatide and lixisenatide is not known to the authors.

In a study, Czock et al. [50] evaluated the impact of severe renal impairment and ESRD on the PK of peptides and proteins. The authors indicated that the molecular size could be a predictor of the effect of renal impairment on the peptides and proteins. Based on their analysis, the authors noted a continuous nonlinear relationship between molecular weight (1.02 to 150 kDa) and AUC in patients with severe renal impairment and ESRD. Based on their study, Czock et al. concluded that changes in the AUC for drugs with a molecular weight below 50 kDa in severe renal impairment and ESRD should be expected.

On the other hand, there are instances when RI (even severe) had no impact on the PK of a peptide. Jacobsen et al. [51] evaluated the impact of renal impairment on the PK of liraglutide. Liraglutide is a recombinant, acylated analog of human GLP-1. Native GLP-1 is a 30 amino acid peptide produced by the cleavage of the transcription product of the preproglucagon gene [52]. The study indicated that renal impairment (mild, moderate, and severe) had no impact on the PK of liraglutide. Similarly, no impact of renal impairment was observed on the PK of insulin degludec [43].

It should be noted that three approved ADCs have a peptide as a payload. These ADCs are Blenrep (belantamab mafodotin-blmf), Padcev (enfortumab vedotin-ejfv), and Polivy (polatuzumab vedotin-piiq). For Blenrep and Polivy, the impact of severe RI or ESRD on the PK of these ADCs was not known. However, mild or moderate RI had an impact on the PK of Blenrep. On the other hand, no difference in the PK of Polivy was noted between patients with mild or moderate RI and normal renal function. Mild, moderate, and severe RI impairment had no impact on the PK of Padcev.

Based on the published literature and the information obtained from FDA PIs, it appears that renal impairment (at least moderate and severe) and ESRD may have a clinically meaningful impact on the PK of peptides, especially in severe renal impairment, and this will require dose adjustment in this patient population. As noted above, the dilemma is that for some peptides even severe renal impairment has no impact on the PK of peptides. Therefore, a rule of thumb or a generalized rule cannot be adopted. Therefore, renal impairment studies should be conducted for peptides, especially in severe renal impairment. One can initiate a renal impairment study in subjects with severe renal impairment and based on the results may proceed to conduct a study in subjects with moderate and/or mild renal impairment.

## 9. Hepatic Impairment (Table 3)

Like renal impairment, dose adjustment for a peptide should be considered if a substantial increase or reduction in exposure of a peptide is noted in patients with hepatic impairment. Out of 16 peptides evaluated in this review, no information was available on the impact of hepatic impairment on the PK of 8 peptides (Table 2). For some peptides such as Vyleesi (bremelanotide), Tresiba (insulin degludec), and Ninlaro (ixazomib), hepatic impairment (mild, moderate, and severe) did not cause any alteration in the PK. For all these three peptides, the AUC increased in subjects with hepatic impairment by a very small margin (Table 2).

In a study, Flint et al. [53] evaluated the impact of hepatic impairment on the PK of liraglutide. Both C_max_ and AUC in subjects with mild, moderate, and severe hepatic impairment were lower than those in healthy subjects, the lowest being in subjects with severe hepatic impairment. The clearance of liraglutide was 1.9-fold higher in subjects with severe hepatic impairment than healthy subjects.

The observation of Flint et al. is in sharp contrast to the small molecules. A general tendency with small molecules is that with the increasing severity of hepatic impairment, the C_max_ and AUC increase and clearance decreases. Although hepatic impairment slightly increased the exposure of Vyleesi, Tresiba, and Ninlaro, for liraglutide, the observation of Flint et al. was quite reverse. In fact, the observation of Flint et al. was in line with an FDA survey conducted to evaluate the impact of hepatic impairment on the PK of monoclonal antibodies (mAbs) as well as antibody–drug conjugates (ADCs) (54)

Based on the data collected by Sun et al. [54] from the pharmaceutical companies’ submissions to the FDA from 2013 to 2018, the authors noted that there were almost no data for severe HI, limited data for moderate HI, and abundant data for mild HI. A significant decrease in AUC was found for several mAbs or ADCs, and a trend for decreasing AUC was observed for other mAbs. The authors’ overall conclusions were that hepatic impairment might impact the elimination of the mAbs as well as the mAb portion of the ADCs. The peptides are much smaller in size than mAbs or ADCs, but looking at the data as described previously, it seems that there remains a probability that hepatic impairment may impact the PK of peptides.

Flint et al.’s observation that exposure of peptides decreases or clearance increases with the severity of hepatic impairment leads to the importance of hepatic impairment studies for peptides. The reason for this is that safety may not be a concern but the subtherapeutic dose of a peptide may be a concern. For example, the clearance of liraglutide was 1.9-fold higher in subjects with severe hepatic impairment than healthy subjects, and this will require a dose adjustment of liraglutide in patients with severe hepatic impairment. The FDA PI [55], however, indicates that no dose adjustment of liraglutide is needed despite the fact that a 42% decrease in the AUC of liraglutide in patients with severe hepatic impairment as compared to healthy subjects was noted.

At the moment, there are not many studies available that have evaluated the impact of hepatic impairment on the PK of peptides. However, considering the characteristics of peptides, that is, peptides are not metabolized by the cytochrome P-450 system, it is highly unlikely that hepatic impairment (even severe) will produce any clinically meaningful impact on the PK of peptides. On the other hand, looking at the observations of Flint et al. and FDA survey, it appears that there may be instances where hepatic impairment of a particular peptide will result in reduced exposure, especially in severe hepatic impairment, to the extent that dose adjustment will be needed.

## 10. Drug Interaction Studies (Table 3)

With the exception of Scenesse (afamelanotide), drug interaction studies of peptides were reasonably well conducted and described in the package inserts. If a dedicated drug interaction study was not conducted then at least in vitro studies were conducted to determine the impact of metabolizing enzymes and transporters on peptides. In some cases, direct drug interaction studies with small molecules were conducted, and the results were variable. For example, drug interaction studies of lixisenatide were conducted with acetaminophen, oral contraceptives, warfarin, atorvastatin, digoxin, and ramipril. The results were variable and changes in C_max_ and AUC were time-dependent (Table 2). Coadministration of ixazomib with rifampin (a strong CYP3A inducer) decreased ixazomib C_max_ and AUC by 54% and 74%, respectively. On the other hand, coadministration of ixazomib with clarithromycin (strong CYP3A inhibitor) and strong CYP1A2 inhibitors did not result in a clinically meaningful change in the systemic exposure of ixazomib (Table 2). It is worth noting that a strong inducer substantially increased the exposure of ixazomib, yet a strong inhibitor did not alter the PK of ixazomib. This, however, does not mean that an enzyme inhibitor of the cytochrome P-450 system will not have an impact on the PK of other peptides.

In a review article, Mahmood and Green [56] noted that some small molecules may not have any impact on the PK of monoclonal antibodies but antibodies can induce or inhibit the cytochrome P-450 system, which may lead to a significant impact on the PK of a small molecule. Although data are not available at this time, the observation of Mahmood and Green with monoclonal antibodies can also be extended to peptides. Therefore, conducting drug–drug interaction studies to evaluate the impact of peptides on the small molecule when given with a peptide is also important and should be done with rigor.

## 11. Immunogenicity (Table 4)

The FDA package inserts indicate that immunogenicity studies were conducted for most of the peptides (evaluated in this review) (Table 4). No information on the immunogenicity of 7 peptides out of 16 evaluated in this review was available in the FDA PI. Overall, immunogenicity incidence for peptides does not appear to be high (Table 4). The incidence of neutralizing antibodies should be investigated, and its impact on the PK, efficacy, and safety should be evaluated.

## 12. Pregnancy (Table 4)

There is no dedicated evaluation of the impact of pregnancy on the PK, efficacy, and safety of peptides. The package inserts of peptides provide a general statement: “There are no data on the use of peptides in pregnant women to evaluate for drug-associated risk, major birth defects and miscarriage, or adverse maternal or fetal outcomes”. It should be noted that the information on embryo–fetal harm comes from rat studies (embryo–fetal development) at higher or equal to human recommended dose (for example, lixisenatide and macimorelin). Overall, there is no conclusive information in the FDA package inserts related to pregnancy and the use of peptides in pregnant women.

The FDA seems to use cautionary language for the use of peptides in pregnant women, but the Agency does not outright contraindicate peptides in pregnant women. This is, however, a serious issue because a lot of pregnant women will be taking one of these peptides without knowing its harmful effect on the fetus or on their own health. Studies must be conducted in this direction.

## 13. Lactation (Table 4)

There is no dedicated study about the appearance of peptides in human milk. The package inserts of ADCs provide a general statement: “There are no data on the presence of peptides in human milk, the effects on the breastfed child, or milk production”. Some information regarding the excretion of milk comes from animal data, mainly from rats. For example, excretion of etelcalcetide in rat milk was assessed following a single intravenous dose of (14C)-etelcalcetide in lactating rats at the human clinical dose of 15 mg three times per week. (14C)-Etelcalcetide was present in the milk at concentrations similar to plasma. A study in lactating rats showed low (9.4%) transfer of lixisenatide and its metabolites into milk. In lactating rats, insulin degludec was present in milk at a concentration lower than that in plasma. These observations warrant the evaluation of peptide excretion in human milk.

## 14. Conclusions

This review summarizes the impact of intrinsic and extrinsic factors on the PK of peptides. It is well known that intrinsic and extrinsic factors generally have a substantial impact on the PK of small molecules, but the impact of these factors has not been thoroughly studied for macromolecules.

From this review, it is evident that both intrinsic and extrinsic factors do have an impact on the PK of peptides. However, the studies to determine the true impact of these factors on peptides are not extensive or rigorous. The current package inserts of the FDA on peptides are lacking important information on the impact of the intrinsic and extrinsic factors on the PK of peptides.

The impact of age, race, and sex on the PK of peptides may be clinically relevant and should be evaluated. Considering that small peptides are eliminated by glomerular filtration, a PK study in the elderly, over 65 years of age, is especially warranted. Furthermore, it is of utmost importance that the PK of those peptides which will be given to pediatrics be rigorously evaluated for rational dosing of peptides in this age group.

Obesity is on the rise both in adults and children, especially in children. Therefore, obesity is an important covariate; where possible, the impact of obesity on the PK of peptides should be evaluated.

Small peptides are mainly excreted by glomerular filtration; hence, it is anticipated that renal function will have an impact on the PK of peptides. Several studies of peptides have been conducted to evaluate the impact of renal impairment on the PK of peptides. In renal impairment, drug doses are adjusted based on the magnitude of the exposure. Generally, the exposure of a drug increases with the severity of the renal impairment. In the case of some peptides, no dose adjustment was recommended by the FDA in subjects with renal impairment despite the fact that the exposure of the particular peptide was much higher in subjects with renal impairment than in subjects with normal renal function. For example, the PK study indicated that the AUC of Tymlos (abaloparatide) and Adlyxin (lixisenatide) increased by 2.1-fold and 124% in patients with severe renal impairment, respectively, yet dose adjustment was not recommended by the FDA. Even if the therapeutic index is wide for peptides, one should not give unnecessarily higher doses than needed in a patient population. Therefore, dose adjustment of peptides in subjects with renal impairment based on PK data must be considered.

Like renal impairment, dose adjustment for a peptide should be considered if a substantial exposure (higher or lower) of a peptide is noted in patients with hepatic impairment. At the moment, there are not many studies available that have evaluated the impact of hepatic impairment on the PK of peptides. It appears that unlike renal impairment, where exposure of peptides increases with the severity of the disease (reconciles well with small molecules), the exposure of peptides decreases with the severity of hepatic impairment.

Flint et al.’s study [53] indicated that the exposure of liraglutide is lower in patients with hepatic impairment than in healthy subjects, the lowest being in subjects with severe hepatic impairment. The clearance of liraglutide was 1.9-fold higher in subjects with severe hepatic impairment than in healthy subjects. Despite this increase in clearance of liraglutide, the FDA recommended that no dose adjustment is needed in patients with hepatic impairment. Although hepatic impairment slightly increased the exposure of Vyleesi, Tresiba, and Ninlaro, for liraglutide, the observation was quite reverse. From the currently available data on hepatic impairment, there is uncertainty to reach a definite conclusion. In some cases, there may be a clinically insignificant increase in a peptide in patients with hepatic impairment, but from the observation of liraglutide, it is possible for there to be a substantial decrease in the exposure which will require dose adjustment. Higher clearance or lower exposure of a peptide will lead to the subtherapeutic dose of a peptide. This is an important issue and must be seriously considered.

At the moment, there is very little information available on the impact of renal and hepatic impairment on the PK of peptides. Therefore, such studies must be conducted, and where necessary, the dose of a peptide must be adjusted. One may start a PK study with severe renal and/or hepatic impairment and, based on the results, decide to conduct studies in moderate and/or mild hepatic impairment. The results of the studies should be used for dosing purposes. A change of >30% in exposure (mainly AUC) in renal and/or hepatic impairment as compared to healthy subjects requires consideration for dose adjustment of peptides. However, a serious dilemma will occur if the renal impairment requires lowering of the peptide dose whereas hepatic impairment requires increasing the dose. In the opinion of the authors, regular PK studies of peptides in patients with hepatic and renal impairment should be conducted unless a clear picture emerges.

The impact of pregnancy on the PK of peptides remains unknown. Similarly, the excretion of peptides in mother’s milk is not known. Both of these are important factors and should be pursued.

In the absence of dedicated clinical pharmacology, attempts were made to determine the impact of intrinsic and extrinsic factors on the PK of peptides through population pharmacokinetics (POPPK). While POPPK provides a reasonable approximation of PK parameters, it should be recognized that in order to detect the influence of covariates on PK parameters from POPPK, the sample size should be adequate. For peptides, it appears that in many cases such as age, gender, severe hepatic or renal impairment, and drug–drug interaction studies, the sample size was simply not sufficient to detect the impact of intrinsic and extrinsic factors on the PK of peptides and yet conclusions were drawn. The subsequent conclusion that the impacts of intrinsic and extrinsic factors are unknown or there were not enough data to draw any conclusion is not a rational approach.

Theoretically, it may sound reasonable not to conduct dedicated clinical pharmacology studies for renal and hepatic impairment and drug interaction due to the physicochemical properties of peptides. However, due to the lack of extensive information and the existence of very few studies regarding renal and hepatic impairment and drug interaction, it is important that dedicated clinical pharmacology studies be conducted for these factors. From these dedicated studies, if it is established that dedicated clinical pharmacology studies are not needed, then POPPK-based analysis should be acceptable. Model-based conclusions require good data and well-established reliable models. The underlying assumptions play a vital role in dictating the potential accuracy of a model. Meaningless number crunching is not in the interest of public health, and the regulatory agencies should recognize the shortcomings of models in a biological system.

The ultimate objective of the study of intrinsic and extrinsic factors is to determine the real magnitude of the impact of these factors in terms of exposure (C_max_ and AUC) so that the dose of a drug can be adjusted or optimized. POPPK may provide the direction of impact (positive or negative or none), but the magnitude of the impact may not be accurate, resulting in a suboptimum or toxic dose. Therefore, dedicated studies of these factors are warranted.

In short, peptides are very useful therapeutic agents for the treatment of a wide variety of diseases. A more stringent approach for the development and approval of peptides is needed. The impact of intrinsic and extrinsic factors should be evaluated rigorously so that an optimum dose can be selected for a patient or a patient population.

## Figures and Tables

**Table 1:** FDA-approved peptides and their characteristics.

Trade Name (Active Ingredient)	FDA Approval	Indication	Target Receptor
Imcivree (setmelanotide)	2020	Obesity	Melanocortin-4 receptor
Scenesse (afamelanotide)	2019	Erythropoietic protoporphyria	Melanocortin 1 receptor
Vyleesi (bremelanotide injection)	2019	Hypoactive sexual desire disorder	Melanocortin receptors
Lutathera (lutetium Lu 177 dotatate)	2018	Gastroenteropancreatic neuroendocrine tumors	Somatostatin receptor
Giapreza (angiotensin II)	2017	Septic shock, diabetes mellitus, and acute renal failure	Type-1 angiotensin II receptor
Macrilen (macimorelin)	2017	Diagnosis of adult growth hormone deficiency	Growth hormone secretagogue receptor type 1
Ozempic (semaglutide)	2017	Diabetes type (II)	Glucagon-like peptide 1 receptor
Parsabiv (etelcalcetide)	2017	Secondary hyperparathyroidism in adult chronic kidney disease	Calcium-sensing receptor
Trulance (plecanatide)	2017	Chronic idiopathic constipation	Guanylate cyclase-C
Tymlos (abaloparatide)	2017	Anabolic agent	Parathyroid hormone 1 receptor
Adlyxin (lixisenatide)	2016	Diabetes type (II)	Glucagon-like peptide 1 receptor
Tresiba (insulin degludec)	2015	Diabetes type (II)	Glucagon-like peptide 1 receptor
Ninlaro (ixazomib)	2015	Multiple myeloma	Beta 5 subunit of the 20S proteasome
**Peptides in Antibody–Drug Conjugates**				
Blenrep (belantamab mafodotin-blmf)	2020	Relapsed or refractory multiple myeloma	B-cell maturation antigen (BCMA)
Polivy (polatuzumab vedotin-piiq)	2019	Refractory diffuse large B-cell lymphoma	CD79b receptor expressed in mature B-cells
Padcev (enfortumab vedotin-ejfv)	2019	Urothelial cancers	Nectin-4 receptor

**Table 2:** Impact of age, sex, and race on the pharmacokinetics of peptides.

Peptides	Age (Young vs. Elderly)	Sex	Race	Pediatrics
Imcivree (setmelanotide)	The effect of age 65 years or older not known.	No clinically significant differences in the PK.	No information provided. It should be interpreted as unknown.	AUC and C_max_ were 100% and 92% higher in children 6 to <12 years of age than adults.
Scenesse (afamelanotide)	No information provided.	No information provided.	No information provided.	No information provided.
Vyleesi (bremelanotide injection)	No information provided.	No information provided.	No information provided.	No information provided.
Lutathera (lutetium Lu 177 dotatate)	The response rate and number of patients with a serious adverse event were similar to those of younger subjects.	No information provided.	No information provided.	No information provided.
Giapreza (angiotensin II)	No significant difference in safety or efficacy between patients <65 and >65 years of age.	No significant difference in PK.	No information provided.	No information provided.
Macrilen (macimorelin)	Not enough patients to evaluate the difference in response between patients <65 and >65 years of age.	No information provided.	No information provided.	No information provided.
Ozempic (semaglutide)	No clinically meaningful effect on the PK. No significant difference in safety or efficacy between patients <65 and >65 years of age.	No clinically meaningful effect on the PK.	No clinically meaningful effect on the PK. African Americans, Asians, and Hispanics.	No information provided.
Parsabiv (etelcalcetide)	No influence on the PK (age = 20–93 years).	No influence on the PK.	No influence on the PK.	No information provided.
Trulance (plecanatide)	Not enough patients to evaluate the difference in response between patients <65 and >65 years of age.	No information provided.	No information provided.	Contraindicated in pediatric patients <6 years of age. Avoid use of Trulance in patients 6 years to <18 years of age.
Tymlos (abaloparatide)	No age-related differences in the PK were observed in postmenopausal women 49 to 86 years of age.	Not applicable. Postmenopausal women with osteoporosis.	No impact on the PK.	No information provided.
Adlyxin (lixisenatide)	No difference in safety and efficacy between patients <65 and >65 years of age.	No meaningful effect on the PK.	No meaningful effect on the PK.	No information provided.
Tresiba (insulin degludec)	No difference in the PK and PD between patients <65 and >65 years of age.	No clinically meaningful effect on the PK.	No statistically significant differences in the PK and PD of Tresiba between African Americans, White Hispanics, and non-Hispanics.	After adjusting for body weight, the total exposure of Tresiba at steady state was independent of age (1 to <18 years of age).
Ninlaro (ixazomib)	No clinically meaningful effect on clearance (CL) (age = 23–91 years). No difference in safety and efficacy between patients <65 and >65 years of age.	No clinically meaningful effect on CL.	No clinically meaningful effect on CL.	No information provided.
**Peptides in Antibody–Drug Conjugates**				
Blenrep (belantamab mafodotin-blmf)	No clinically significant differences in the PK.	No clinically significant differences in the PK.	No clinically significant differences in the PK.	No information provided.
Polivy (polatuzumab vedotin-piiq)	No clinically significant differences in the PK.	No clinically significant differences in the PK.	No clinically significant differences in the PK.	No information provided.
Padcev (enfortumab vedotin-ejfv)	No clinically significant differences in the PK.	No clinically significant differences in the PK.	No clinically significant differences in the PK.	No information provided.

**Table 3:** Drug interaction and impact of hepatic and renal impairment on the pharmacokinetics of peptides.

Peptides	Hepatic Impairment (HI)	Renal Impairment (RI)	Drug Interaction
Imcivree (setmelanotide)	No information provided.	A 19% higher AUC in patients with mild RI than patients with normal renal function. Not for use in patients with moderate, severe RI, and end-stage renal disease (ESRD).	In vitro study of drug–drug interactions indicated that setmelanotide has low potential for PK drug–drug interactions related to cytochrome P450 (CYP 450) and transporters. No clinical studies evaluating the drug–drug interaction potential of setmelanotide were conducted.
Scenesse (afamelanotide)	No information provided.	No information provided.	No information provided
Vyleesi (bremelanotide injection)	Following a single SC dose of Vyleesi, the AUC increased 1.2-fold and 1.7-fold, with mild and moderate HI. No information available for severe HI.	Following a single SC dose of Vyleesi, the AUC increased by 1.2-fold, 1.5-fold, and 2-fold in patients with mild, moderate, and severe RI.	Vyleesi may slow gastric emptying and thus has the potential to reduce the rate and extent of absorption of concomitantly administered oral medications. Vyleesi reduced the C_max_ and AUC for naltrexone and indomethacin by 40–60% and 20 to 40%, respectively.
Lutathera (lutetium Lu 177 dotatate)	No dose adjustment was recommended for patients with mild to moderate HI (reason for this is not known). No information on severe HI.	No dose adjustment was recommended for patients with mild to moderate RI (reason for this is not known). No information on severe RI or ESRD.	The nonradioactive form of lutetium is not an inhibitor or inducer of cytochrome P450 enzymes in vitro (1A2, 2B6, 2C9, 2C19, or 2D6). It is also not an inhibitor of P-glycoprotein, BCRP, OAT1, OAT3, OCT2, OATP1B1, OATP1B3, or OCT1 in vitro.
Giapreza (angiotensin II)	No PK study was conducted with Giapreza because its clearance is not dependent on hepatic function.	No PK study was conducted with Giapreza because its clearance is not dependent on renal function.	Concomitant use of angiotensin-converting enzyme (ACE) inhibitors may increase the response to Giapreza, whereas concomitant use of angiotensin II blockers may decrease the response to Giapreza.
Macrilen (macimorelin)	No information provided.	No information provided.	Coadministration of Macrilen with drugs that prolong the QT interval may lead to development of torsade de pointes-type ventricular tachycardia. Coadministration of a strong CYP3A4 inducer with Macrilen may reduce the plasma Macrilen concentrations.
Ozempic (semaglutide)	No dose adjustment of Ozempic is needed for patients with mild, moderate, and severe HI. The source of this information is not known.	No dose adjustment of Ozempic is needed for patients with mild, moderate, and severe RI. The source of this information is not known.	Ozempic causes a delay of gastric emptying and thereby has the potential to impact the absorption of concomitantly administered oral medications.
Parsabiv (etelcalcetide)	No information provided.	No information provided.	Etelcalcetide did not inhibit or induce CYP450 enzymes and is not a substrate of CYP450 enzymes. Etelcalcetide was not a substrate of efflux and uptake transporter of P-glycoprotein (Pgp).
Trulance (plecanatide)	No information provided.	No information provided.	In vitro studies indicated that plecanatide and its active metabolite do not inhibit or induce CYP3A4. Plecanatide and its active metabolite are neither substrates nor inhibitors of Pgp.
Tymlos (abaloparatide)	No information provided.	A PK study indicated that Cmax and AUC of Tymlos increased by 1.4- and 2.1-fold, respectively, in subjects with severe RI as compared to subjects with normal renal function. No dosage adjustment is required for patients with mild, moderate, or severe RI.	No specific drug–drug interaction studies were performed. In vitro studies indicated that Tymlos does not inhibit or induce CYPP450 enzymes.
Adlyxin (lixisenatide)	No PK study was performed in patients with HI. HI is not expected to affect the PK of lixisenatide.	Compared to healthy subjects, plasma Cmax and AUC of lixisenatide increased by 60%, 42%, and 83% and 34%, 69%, and 124% in subjects with mild, moderate, and severe RI, respectively. No dose adjustment in patients with RI was recommended.	Drug interaction studies of lixisenatide were conducted with acetaminophen, oral contraceptives, warfarin, atorvastatin, digoxin, and ramipril. The results were variable and changes in Cmax and AUC were time-dependent.
Tresiba (insulin degludec)	No difference in the PK of Tresiba following an SC dose of 0.4 units/kg was noted between healthy subjects and subjects with HI (mild, moderate, and severe).	The PK of Tresiba following an SC dose of 0.4 units/kg was studied in subjects with mild, moderate, and severe RI. Total AUC and Cmax were about 10–25% and 13–27% higher, respectively, in subjects with mild to severe RI. In subjects with ESRD, the exposure of Tresiba was similar to subjects with normal renal function.	A number of medications affect glucose metabolism and may require insulin dose adjustment and close monitoring. The package insert identifies several classes of drugs that may produce clinically significant drug interactions with Tresiba. Drugs that may increase the risk of hypoglycemia (for example, antidiabetic agents, ACE inhibitors, angiotensin II receptor blocking agents, monoamine oxidase inhibitors, and salicylates). Drugs that may decrease the blood glucose lowering effect of Tresiba (for example, atypical antipsychotics, protease inhibitors, sympathomimetic agents, and thyroid hormones). Drugs that may increase or decrease the blood glucose lowering effect of Tresiba (for example, alcohol, beta-blockers, clonidine, and lithium salts). Drugs that may blunt signs and symptoms of hypoglycemia (for example, beta-blockers, clonidine, guanethidine, and reserpine).
Ninlaro (ixazomib)	In patients with moderate or severe HI, the mean AUC increased by 20% as compared to patients with normal hepatic function.	The PK of ixazomib was similar in patients with normal renal function and in patients with mild or moderate RI. Mean AUC was 39% higher in patients with severe RI or in patients with ESRD requiring dialysis as compared to patients with normal renal function.	Coadministration of Ninlaro with rifampin (a strong CYP3A Inducer) decreased ixazomib Cmax and AUC by 54% and 74%, respectively. Coadministration of Ninlaro with clarithromycin (strong CYP3A Inhibitors) and strong CYP1A2 inhibitors did not result in a clinically meaningful change in the systemic exposure of ixazomib. Ninlaro is not expected to produce drug–drug interactions via CYP inhibition or induction. Ixazomib is a low-affinity substrate of P-gp.
Blenrep (belantamab mafodotin-blmf)	Mild HI had no impact on the PK of Blenrep. The impact of moderate and severe HI on the PK of Blenrep is not known.	Mild or moderate RI had impact on the PK of Blenrep. The impact of severe RI or ESRD with or without dialysis on the PK of Blenrep is not known.	Monomethyl auristatin F (MMAF), a payload, is a substrate of organic anion transporting polypeptide (OATP)1B1 and OATP1B3, multidrug resistance-associated protein (MRP)1, MRP2, MRP3, bile salt export pump (BSEP), and a possible substrate of P-gp.
Polivy (polatuzumab vedotin-piiq) 2019	In patients with mild HI, the PK of monomethyl auristatin E (MME) was similar between patients with normal hepatic function but unconjugated MME was higher by 40% in subjects with HI. The impact of moderate and severe hepatic impairment or liver transplantation on the PK of MME is not known.	No difference in the PK of conjugated and unconjugated MME was noted between patients with mild or moderate RI and normal renal function. The impact of severe RI and in patients with ESRD on the PK of MME is not known.	No dedicated drug–drug interaction clinical study of Polivy was conducted. POPPK analysis indicated that concomitant rituximab was associated with increased conjugated MMAE AUC by 24% and decreased unconjugated MMAE AUC by 37%.
Padcev (enfortumab vedotin-ejfv) 2019	POPPK study indicated that there was a 48% increase in the AUC of unconjugated MMAE in patients with mild HI as compared to subjects with normal hepatic function. The effect of moderate or severe HI on the PK of Padcev or unconjugated MMAE is not known.	Following 1.2 mg/kg dose of Padcev, mild, moderate, and severe RI impairment had no impact on the PK of Padcev or unconjugated MMAE. The effect of end-stage renal disease with or without dialysis on the PK of Padcev or unconjugated MMAE is not known.	Drug–drug interaction studies of Padcev have not formally been evaluated. Ketoconazole (a strong CYP3A4 inhibitor) increased MMAE Cmax by 25% and AUC by 34%. Rifampin (a strong CYP3A4 inducer) decreased MMAE Cmax by 44% and AUC by 46%.

**Table 4:** Impact of pregnancy, lactation, and immunogenicity on the pharmacokinetics of peptides.

Peptides	Pregnancy	Lactation	Immunogenicity
Imcivree (setmelanotide)	The FDA package insert states “Discontinue Imcivree when pregnancy is recognized unless the benefits of therapy outweigh the potential risks to the fetus”. There are no available data with Imcivree in pregnant women to inform a drug-associated risk for major birth defects and miscarriage, or adverse maternal or fetal outcomes.	Treatment with Imcivree is not recommended for use while breastfeeding. There is no information on the presence of setmelanotide or its metabolites in human milk, the effects on the breastfed infant, or the effects on milk production.	Approximately 61% of adult and pediatric patients who received Imcivree (*n* = 28) were positive for antibodies to Imcivree Lack of decline in Imcivree concentrations to suggest the presence of antidrug antibodies.
Scenesse (afamelanotide)	No information provided.	No information provided.	No information provided.
Vyleesi (bremelanotide injection)	There are not enough data in pregnant women to determine a drug-associated risk of adverse effects, major birth defects, miscarriage, or adverse maternal or fetal outcomes.	There is no information on the presence of bremelanotide or its metabolites in human milk, the effects on the breastfed infant, or the effects on milk production.	No information provided.
Lutathera (lutetium Lu 177 dotatate)	Based on its mechanism of action, Lutathera can cause fetal harm. There are no available data on Lutathera use in pregnant women.	There are no data on the presence of lutetium Lu 177 dotatate in human milk or its effects on the breastfed infant or milk production.	No information provided.
Giapreza (angiotensin II)	There are not enough data in pregnant women to determine a drug-associated risk of adverse effects.	It is not known whether Giapreza is present in human milk. No data are available on the effects of Giapreza on the breastfed child or the effects on milk production.	No information provided.
Macrilen (macimorelin)	There are no available data with Macrilen use in pregnant women to inform a drug-associated risk for adverse effects.	There are no data on the presence of macimorelin in human milk, the effects on the breastfed infant, or the effects on milk production.	No information provided.
Ozempic (semaglutide)	There are limited data with semaglutide use in pregnant women to assess drug-associated risk for adverse effects. Ozempic should be used during pregnancy only if the potential benefit justifies the potential risk to the fetus.	There are no data on the presence of semaglutide in human milk, the effects on the breastfed infant, or the effects on milk production.	Across the placebo- and active-controlled glycemic control trials, 32 (1.0%) Ozempic-treated patients developed antidrug antibodies.
Parsabiv (etelcalcetide)	There are no available data on the use of Parsabiv in pregnant women.	There are no data regarding the presence of Parsabiv in human milk or effects on the breastfed infant or on milk production.	Patients (7.1%) with secondary hyperparathyroidism treated with etelcalcetide for up to 6 months tested positive for binding anti-etelcalcetide antibodies.
Trulance (plecanatide)	There are not enough data in pregnant women to assess any drug-associated risks for major birth defects and miscarriage. Since plecanatide and its active metabolite are negligibly absorbed following oral administration, maternal use is not expected to result in fetal exposure to the drug.	There is no information regarding the presence of plecanatide in human milk or its effects on milk production or the breastfed infant.	No information provided.
Tymlos (abaloparatide)	Tymlos is not indicated for use in females of reproductive potential. There are no human data with Tymlos use in pregnant women to assess any drug-associated risks.	There is no information on the presence of abaloparatide in human milk, the effects on the breastfed infant, or the effects on milk production	Following 18 months of Tymlos treatment, 49% of subjects developed anti-abaloparatide antibodies; of these, 68% developed neutralizing antibodies to abaloparatide.
Adlyxin (lixisenatide)	There are not enough data with lixisenatide in pregnant women to assess the risk of major birth defects and miscarriage.	There is no information regarding the presence of Adlyxin in human milk, the effects on the breastfed infant, or the effects on milk production.	Seventy percent of patients exposed to lixisenatide tested positive for anti-lixisenatide antibodies. No information regarding the presence of neutralizing antibodies is available.
Tresiba (insulin degludec)	There are no available data with Tresiba in pregnant women about drug-associated risk for major birth defects and miscarriage.	There are no data on the presence of insulin degludec in human milk, the effects on the breastfed infant, or the effects on milk production.	In adult type 1 diabetic patients, 95.9% were positive for anti-insulin antibodies (AIA) at least once during the studies, including 89.7% that were positive at baseline. In studies of type 2 diabetic patients, 31.5% of patients were positive for AIA at least once during the studies, including 14.5% that were positive at baseline.
Ninlaro (ixazomib)	Ninlaro can cause fetal harm when administered to a pregnant woman. There are no human data available regarding the effect of Ninlaro on pregnancy or development of the embryo or fetus.	It is not known whether Ninlaro or its metabolites are present in human milk.	No information provided.
Polivy (polatuzumab vedotin-piiq),	There are no available data on the use of Polivy in pregnant women to evaluate for drug-associated risk, risk of major birth defects, and miscarriage.	There are no data on the presence of Polivy in human milk or the effects on the breastfed child or milk production.	Across all studies, 8 out of 134 (6%) patients were tested positive for antibodies against Polivy at one or more post-baseline time points.
Blenrep (belantamab mafodotin-blmf),	Based on its mechanism of action, Blenrep can cause fetal harm when administered to a pregnant woman, because it contains a genotoxic compound (the microtubule inhibitor MMAF) and it targets actively dividing cells. There are no available data on the use of Blenrep in pregnant women to evaluate for drug-associated risk.	There are no data on the presence of belantamab mafodotin-blmf in human milk or the effects on the breastfed child or milk production.	In clinical studies of Blenrep, 2/274 patients (<1%) tested positive for anti-Blenrep antibodies after treatment.
Padcev (enfortumab vedotin-ejfv),	There are no available data on the use of Padcev in pregnant women to evaluate for drug-associated risk, major birth defects, and miscarriage.	There are no data on the presence of Padcev in human milk or the effects on the breastfed child or milk production.	Out of 365 patients evaluated for immunogenicity to Padcev, 4 patients (1%) were found to be transiently positive for anti-Padcev antibody.
